# Exploration of Endophytic Fungal Communities in Three *Hypericum* Species: Molecular Identification and Evaluation of Antibacterial Activity Against Clinically Relevant Pathogens

**DOI:** 10.5812/ijpr-167951

**Published:** 2026-06-28

**Authors:** Fahimeh Nabi, Ahmad Asgharzadeh, Javad Asili, Abolfazl Shakeri

**Affiliations:** 1 Department of Agriculture, Shirvan Branch, Islamic Azad University, Shirvan, Iran; 2 Department of Pharmacognosy, School of Pharmacy, Mashhad University of Medical Sciences, Mashhad, Iran; 3 Pharmacological Research Center of Medicinal Plants, Mashhad University of Basic Medical Sciences, Mashhad, Iran

**Keywords:** Hypericum, Endophytic Fungi, Secondary Metabolites, Antimicrobial Activity

## Abstract

**Background:**

Endophytic fungi, which inhabit internal plant tissues without causing apparent disease, have emerged as rich sources of bioactive secondary metabolites. *Hypericum* species are well-known medicinal plants with diverse pharmacological properties; however, the endophytic fungi associated with these species remain largely unexplored.

**Objectives:**

This study aimed to isolate and molecularly identify endophytic fungi associated with *Hypericum helianthemoides*, *H. scabrum*, and *H. perforatum*, and to evaluate the antibacterial activity of their extracts against selected pathogenic bacteria.

**Methods:**

Endophytic fungi were isolated from the root and stem tissues of three *Hypericum* species. Molecular identification was performed by amplifying and sequencing the internal transcribed spacer (ITS) and large subunit (LSU) regions of the rRNA gene. The antibacterial activity of the fungal extracts was assessed by determining the minimum inhibitory concentration (MIC) and minimum bactericidal concentration (MBC) values against *Staphylococcus aureus*, *S. epidermidis*, *Bacillus subtilis*, *Pseudomonas aeruginosa*, and *Escherichia coli*.

**Results:**

A total of 20 fungal isolates were obtained, mainly belonging to the phylum Ascomycota, with one isolate (Absidia sp.) belonging to Zygomycota. *H. perforatum* harbored the highest number of isolates (40%). Most isolates were newly reported endophytes of *Hypericum* species. The dominant genera were *Fusarium* and Alternaria. More than half of the isolates exhibited antibacterial activity, particularly against Gram-positive bacteria, whereas only three species were active against Gram-negative strains. *Cladosporium subglobosum* demonstrated the most potent antibacterial effect, with MIC and MBC values of 3.125 μg/mL.

**Conclusions:**

This study highlights the diversity and antimicrobial potential of endophytic fungi associated with *Hypericum* species. These findings suggest that such endophytes may represent promising sources of novel bioactive metabolites with potential pharmaceutical applications.

## 1. Background

There are approximately 484 recognized species in the Hypericaceae family, organized into 36 taxonomic sections and encompassing tree-like, shrub-like, and herbaceous growth forms (1). Although members of this family are distributed across most regions worldwide, they are uncommon in tropical low-altitude areas, arid deserts, and high-latitude polar climates (2). Owing to its importance in ethnomedicine and ornamental production, the genus *Hypericum* is of considerable interest (3). *H. helianthemoides* is distinguished within the genus by pharmacological characteristics associated with its essential oil constituents, which have been shown to exhibit antioxidant and antimicrobial properties (4). *H. scabrum* has also received scientific attention because of reports of antioxidant, anti-inflammatory, and cognitive benefits, as well as evidence suggesting potential cytotoxic and pro-apoptotic capacities relevant to cancer research (3). *H. perforatum* is widely used in traditional phytotherapy for conditions such as mild to moderate depression, anxiety, and sleep problems. Modern research has further highlighted its neuroprotective, anti-inflammatory, antiplatelet, antimicrobial, and antioxidant effects (5).

It is important to recognize that the antimicrobial activity attributed to *Hypericum* species may be closely related to their indigenous fungal endophytes. These nonpathogenic fungi, which colonize internal plant tissues, are increasingly recognized as important producers of bioactive metabolites (6). Studies of endophytic communities in *Hypericum* have yielded promising findings regarding their antimicrobial potential (7). Among the *Hypericum* species investigated, *H. perforatum* has received the greatest research attention because of its well-established therapeutic relevance (8). Numerous endophytes, including Colletotrichum, *Penicillium*, and *Aspergillus* species, have been identified in association with these plants (9). These microorganisms produce a wide variety of antimicrobial compounds, including alkaloids and other secondary metabolites (7), which have demonstrated activity against a broad range of pathogens, including bacteria, fungi, and viruses (10). In addition, studies have examined the potential pharmaceutical applications of endophytes residing in *H. perforatum*, particularly their capacity to produce compounds with clinically significant antimicrobial effects (8). Collectively, these findings underscore the complex ecological relationships between *Hypericum* plants and their fungal endophytes, as well as their potential applications in natural defense strategies, drug development, and agricultural advancement.

## 2. Objectives

Examining multiple *Hypericum* species and their associated endophytic fungi provides a valuable opportunity to elucidate previously unrecognized ecological interactions and to identify novel antimicrobial metabolites with potential applications across scientific and practical fields. Although existing findings indicate widespread colonization of *Hypericum* plants by fungal endophytes and suggest potential antimicrobial activities attributable to these microorganisms, research has disproportionately focused on *H. perforatum*. Consequently, several *Hypericum* species remain insufficiently investigated. A major gap in the literature is the lack of comparative analyses assessing patterns of endophytic colonization and fungal diversity among different *Hypericum* species. In addition, it remains unclear whether endophytes from less-studied *Hypericum taxa* exhibit antimicrobial capabilities comparable to those reported for *H. perforatum*. Addressing this knowledge gap is essential not only for improving understanding of plant–endophyte symbioses but also for advancing applications in drug discovery and agricultural biotechnology. Therefore, the present study aimed to molecularly characterize the endophytic fungi isolated from various tissues of *H. helianthemoides*, *H. scabrum*, and *H. perforatum*. The antimicrobial activities of the recovered isolates were further evaluated against *Staphylococcus aureus*, *S. epidermidis*, *Bacillus subtilis*, *Pseudomonas aeruginosa*, and *Escherichia coli*.

## 3. Methods

### 3.1. Plant Collection, Taxonomic Verification, and Herbarium Documentation

Healthy, stress-free specimens were collected at the flowering stage from Quchan, Razavi Khorasan Province, Iran (37°05'45.0"N, 58°30'42.2"E), and their identification was confirmed by Ms. Souzani. Corresponding voucher materials (*H. helianthemoides* 13612, *H. scabrum* 13594, and *H. perforatum* 13571) were subsequently deposited in the Herbarium of the School of Pharmacy, Mashhad University of Medical Sciences, Iran. To obtain whole plants, individuals were carefully excavated using a sterilized spade disinfected with 75% ethanol. Excess soil adhering to the root systems was removed by gently shaking the samples. All specimens were immediately placed in sterile plastic bags and transported on ice to the laboratory for subsequent analyses.

### 3.2. Aseptic Isolation of Endophytic Fungi

Freshly collected plant tissues (roots, stems, leaves, and flowers) were subjected to surface disinfection by immersion in 75% (v/v) ethanol for 1 min, followed by three successive rinses with sterile distilled water to eliminate residual ethanol (11). The tissues were then treated with a 1% (v/v) sodium hypochlorite solution for 1 min and subsequently rinsed three times with sterile distilled water to remove any remaining disinfectant (11, 12). To verify the effectiveness of surface sterilization, two validation procedures were performed. First, aliquots (100 μL) of the final rinse water were plated onto potato dextrose agar (PDA; Merck 110130) supplemented with streptomycin (100 μg/mL) and monitored for microbial growth. Second, surface-sterilized tissue segments were gently pressed onto PDA plates using the imprint method before plating. After sterilization, the tissues were aseptically cut into small fragments using a sterile scalpel and transferred onto PDA containing streptomycin to inhibit bacterial growth. The inoculated plates were incubated at 25 ± 2 °C in darkness for 1 - 2 weeks until fungal growth emerged. Individual fungal colonies were subsequently isolated and transferred to fresh PDA plates to obtain pure cultures.

### 3.3. Extraction and Purification of Fungal Genomic DNA

Fungal isolates were transferred to Erlenmeyer flasks containing potato dextrose broth (PDB; HiMedia M403) and incubated for one week at 25 ± 2 °C under dark conditions. After incubation, the fungal mycelia were collected by filtration through a 200-mesh screen and immediately frozen in liquid nitrogen. The frozen material was then air-dried at 30 ± 2 °C for approximately 1 hour before being pulverized into a fine powder using a sterile mortar and pestle.

For DNA extraction, 200 mg of the ground mycelial powder was mixed with 600 μL of CTAB extraction buffer (2% CTAB, 100 mM Tris-HCl pH 8.0, 20 mM EDTA, and 1.4 M NaCl). The suspension was incubated at 65 °C for 30 minutes in a darkened water bath (Memmert WNB7). After incubation, an equal volume of chloroform-isoamyl alcohol (24:1) was added, and the mixture was centrifuged at 12,000 m/s^2^ for 10 minutes at 25 °C. The resulting aqueous phase was mixed with an equal volume of isopropanol to precipitate genomic DNA and held at −20 °C in the dark for 30 minutes before centrifugation (4 °C; 12,000 m/s^2^; 10 minutes). The DNA pellet was washed with 70% ethanol, rinsed three times with sterile distilled water, and air-dried at 30 ± 2 °C for 1 hour. The final pellet was dissolved in 100 μL of TE buffer (10 mM Tris-HCl pH 8.0 and 1 mM EDTA) and used as the DNA template for downstream analyses (13).

### 3.4. Amplification and BLAST-Based Identification

Polymerase chain reaction (PCR) was used to amplify the ITS and LSU rRNA regions of fungal genomic DNA. Amplification of the ITS region was performed using ITS5 (5´-GGAAGTAAAAGTCGTAACAAGG-3´) as the forward primer and ITS4 (5´-TCCTCCGCTTATTGATATGC-3´) as the reverse primer (14). For the LSU region, LROR (5´-CCCGCTGAACTTAAGC-3´) and LR5 (5´-TCCTGAGGGAAACTTCG-3´) were used as forward and reverse primers, respectively (15). PCR reactions were carried out in a total volume of 12.5 μL, consisting of 1× PCR buffer, 10 - 20 ng of genomic DNA, 0.7 μL of 99.9% DMSO, 0.5 μM of each primer, 25 μM of each dNTP, 1.0 U of Taq DNA polymerase (NEB), and 2 mM MgCl2. Amplifications were performed using a GeneAmp PCR System 9600 (Perkin Elmer, USA) under the following thermal cycling conditions: initial denaturation at 95 °C for 1 minute; 40 cycles of 95 °C for 30 seconds, annealing at 58 °C for 1 minute, and extension at 72 °C for 10 seconds. The amplified sequences were analyzed using the Basic Local Alignment Search Tool for nucleotides (BLASTN) in the National Center for Biotechnology Information (NCBI) database to determine sequence similarity and identity. Sequences exhibiting ≥ 99% identity to type strains in GenBank were assigned at the species level, in accordance with established protocols for fungal species identification using the ITS and LSU loci.

### 3.5. Preparation and Standardization of Bacterial Pathogens for Antibacterial Assays

Pathogenic bacterial strains were obtained from Mashhad University of Medical Sciences and had previously been deposited in the Persian Type Culture Collection (PTCC) and the American Type Culture Collection (ATCC). The selected strains included *S. aureus* (ATCC 25293), *S. epidermidis* (DSMZ 3270), *B. subtilis* (PTCC 1023), *P. aeruginosa* (PTCC 1074), and *E. coli* (PTCC 1330). Cultures were grown in Mueller-Hinton broth (MHB; HiMedia M391) at 37 ± 2 °C under dark conditions for 24 hours using a shaker incubator (3 m/s^2^). After incubation, the bacterial suspensions were centrifuged at 11,200 m/s^2^ for 10 minutes, and the resulting cell pellets were rinsed three times with sterile distilled water. A final bacterial suspension with a concentration of 10^6^ cells/mL was prepared in normal saline, and cell counts were determined using a hemocytometer.

### 3.6. Determination of Minimum Inhibitory and Bactericidal Concentrations of Fungal Extracts

Fungal isolates were cultured in PDB medium for seven days at 25 ± 2 °C under dark conditions with gentle shaking (150 rpm). After incubation, the cultures were filtered through Whatman No. 1 filter paper to separate the mycelial biomass and obtain the culture filtrate. The filtrate was then concentrated under reduced pressure using a rotary evaporator at 40 °C to remove water and obtain a crude bioactive extract. The concentrated extract was further dried under vacuum to constant weight and stored at −20 °C until use. The minimum inhibitory concentration (MIC) of the endophytic fungal extracts was assessed using the broth macro-dilution method in 24-well microplates. Each well received 0.9 mL of varying concentrations of the fungal extract prepared in MHB, followed by the addition of 0.1 mL of a standardized microbial suspension. The initial fungal extract stock was dissolved in Tween 80 at a 6:4 ratio. Sterility and growth controls were included on each plate, consisting of wells containing only MHB as the negative control and wells containing 0.9 mL of MHB plus 0.1 mL of microbial suspension as the positive control. Plates were incubated for 24 hours at 37 ± 2 °C. The standard antibiotics cloxacillin (5 μg/mL) and tobramycin (8 μg/mL) served as positive controls for bacterial growth. Microbial proliferation was monitored using the 2,3,5-triphenyltetrazolium chloride (TTC) assay (Sigma, USA). Specifically, 0.25 mL of a 5 mg/mL TTC solution (Merck) was added to each well, followed by incubation for 3 hours at 37 ± 2 °C. The MIC was recorded as the lowest concentration at which no red formazan color developed. All experiments were performed in duplicate, with each condition tested in triplicate. The minimum bactericidal concentration (MBC), defined as the lowest concentration at which no bacterial colonies were observed, was determined by streaking aliquots from MIC wells and wells with lower concentrations onto soybean casein digest agar plates (HiMedia) and assessing bacterial growth.

### 3.7. Phylogenetic Inference and Evolutionary Analysis

Evolutionary analyses were performed using MEGA11 software. The Neighbor-Joining approach was applied to construct an optimal phylogenetic tree and investigate the evolutionary relationships among the studied taxa (16). Bootstrap analysis with 1,000 replicates was conducted to assess the reliability of the inferred groupings, and the corresponding percentages are indicated at each branch. All phylogenetic trees were scaled, with branch lengths proportional to the calculated evolutionary distances. These distances were estimated using the Maximum Composite Likelihood method and are expressed as the number of nucleotide substitutions per site (17). Ambiguous positions in sequence alignments were excluded using the pairwise deletion strategy. Additional graphical representations were generated using Microsoft Excel.

## 4. Results

4.1. Diversity and Tissue-Specific Colonization Patterns of Endophytic Fungi in *Hypericum* spp.

**Figure 1.**
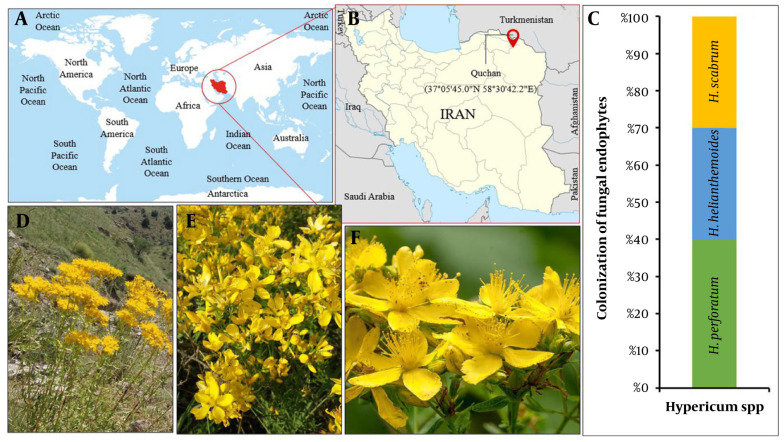
Geographical locations of the collected plant areas are presented as follows: A, The geographical location of Iran, marked in red, on the globe map; B, Geographical locations of the Quchan plant sampling area in Iran; C, A 100% stacked bar chart depicting the abundance percentage of fungal endophytes from different plants; D, *Hypericum scabrum*; E, *H. helianthemoides*; F, *H. perforatum*.

**Figure 2.**
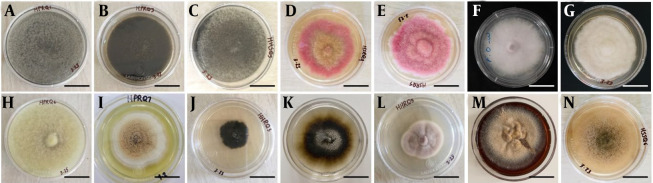
Macroscopic images of plant endophytic fungi. A, Alternaria cerealis; B, *A. sorghi*; C, *A. angustiovoidea*; D, *Fusarium avenaceum*; E, *F. tricinctum*; F, *F. oxysporum*; G, Absidia sp.; H, *Chaetomium subglobosum*; I, *Aspergillus flavipes*; J, *Cladosporium antarcticum*; K, Aureobasidium sp.; L, *Penicillium lilacinum*; M, *Dactylonectria alcacerensis*; N, Phoma macrostoma.

**Figure 3.**
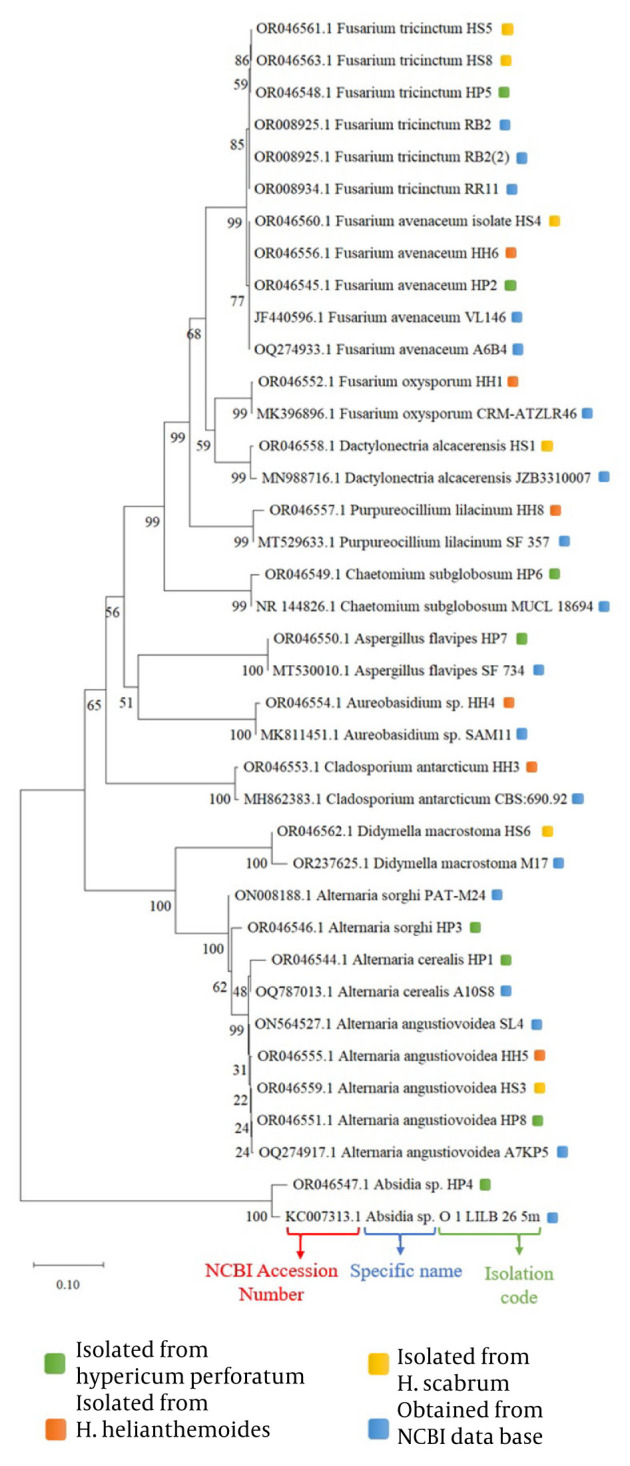
Phylogenetic tree of endophytic fungi from *Hypericum* spp. based on the internal transcribed spacer genomic region, involving 38 nucleotide sequences and a total of 1244 positions in the final dataset.

**Figure 4.**
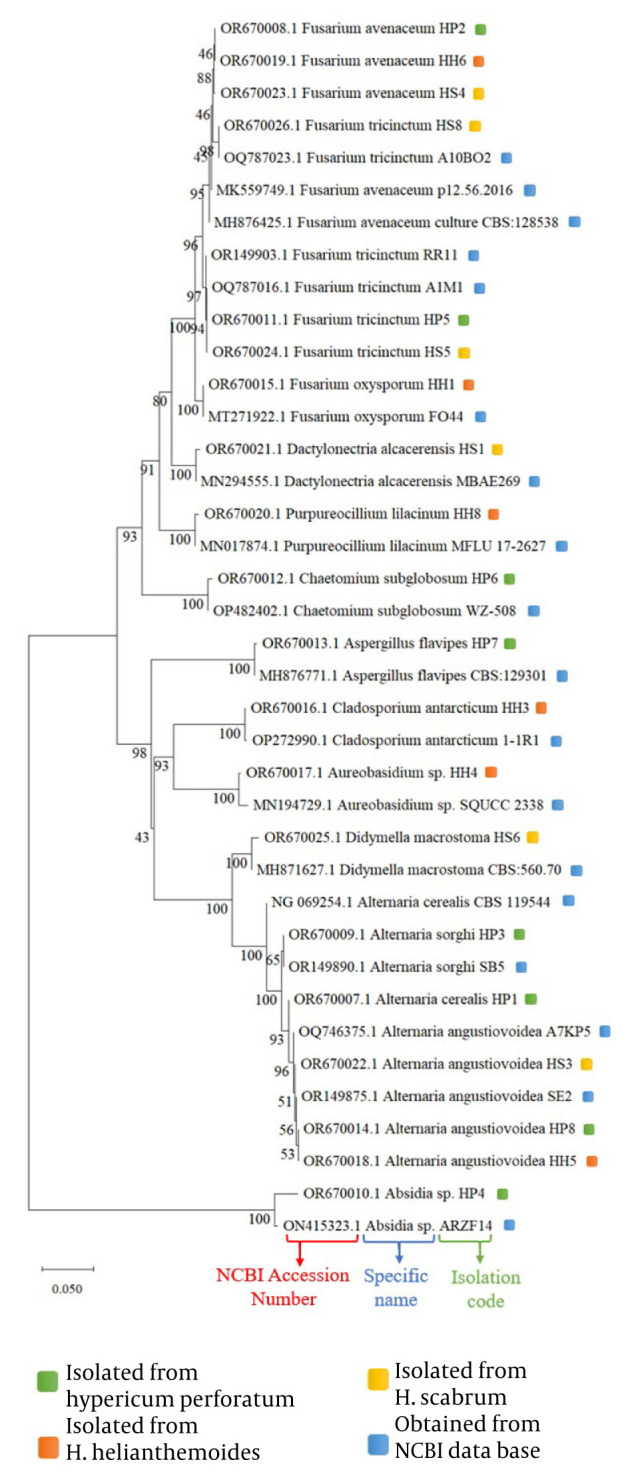
Phylogenetic tree of endophytic fungi from *Hypericum* spp. based on the large subunit rRNA genomic region, involving 38 nucleotide sequences and a total of 1227 positions in the final dataset.

**Figure 5.**
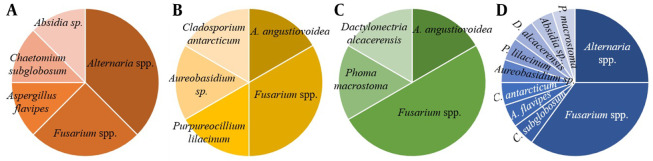
Abundance of identified endophytic fungi from *Hypericum* spp. (D). A, Endophytic fungal abundance in *H. perforatum*; B, Endophytic fungal abundance in *H. helianthemoides*; C, Endophytic fungal abundance in *H. scabrum*.

A total of 20 fungal isolates were recovered from *H. helianthemoides* (Figure 1E), *H. scabrum* (Figure 1D), and *H. perforatum* (Figure 1F), and their identification was confirmed by both morphological and molecular analyses (Figures 2-4). Although all plant tissues were screened, endophytic fungi were detected exclusively in the roots and stems of *Hypericum* species (Table 1). Among the hosts, *H. perforatum* contributed the largest proportion of isolates, accounting for 40% (Figure 1C), whereas *H. helianthemoides* and *H. scabrum* each represented 30% of the isolates. *Fusarium* spp. were the most frequently recovered endophytes, representing 35% of the total isolates (Figure 5D), followed by Alternaria spp., which comprised 25% of all identified fungi. The remaining isolates belonged to other genera, each displaying unique colonization patterns (Table 1; Figure 5). Within *H. perforatum*, Alternaria spp. were the dominant endophytes, accounting for 37.5% of isolates and occurring exclusively in the roots (Table 1; Figure 5A), whereas *Fusarium* spp. represented 25% of the isolates across different tissues (Figure 5A). In *H. helianthemoides* and *H. scabrum*, *Fusarium* species were the most prevalent, with frequencies of 33.33% and 50%, respectively.

**Table 1.** Molecular Identities of the Endophytic Fungi from Different Tissues of *Hypericum* Spp. Sampled in Quchan, Razavi Khorasan Province, Iran (37°05'45.0"N, 58°30'42.2"E) ^a^

Specific Name	NCBI Accession No. (ITS Genomic Region)	ITS Genomic Sequence (bp)	NCBI Accession No. (LSU Genomic Region)	LSU Genomic Sequence (bp)	Plant Host	Tissue
***Alternaria cerealis***	OR046544.1	558	OR670007.1	548	*H. perforatum*	Root
***A. sorghi***	OR046546.1	555	OR670009.1	606	*H. perforatum*	Root
***A. angustiovoidea***	OR046551.1	552	OR670014.1	530	*H. perforatum*	Root
***A. angustiovoidea***	OR046555.1	553	OR670018.1	530	*H. helianthemoides*	Stem
***A. angustiovoidea***	OR046559.1	554	OR670022.1	531	*H. scabrum*	Root
***Fusarium avenaceum***	OR046545.1	539	OR670008.1	582	*H. perforatum*	Root
***F. avenaceum***	OR046556.1	524	OR670019.1	597	*H. helianthemoides*	Root
***F. avenaceum***	OR046560.1	526	OR670023.1	595	*H. scabrum*	Root
***F. tricinctum***	OR046548.1	565	OR670011.1	846	*H. perforatum*	Stem
***F. tricinctum***	OR046561.1	563	OR670024.1	849	*H. scabrum*	Root
***F. tricinctum***	OR046563.1	566	OR670026.1	866	*H. scabrum*	Stem
***F. oxysporum***	OR046552.1	542	OR670015.1	858	*H. helianthemoides*	Root
***Absidia* sp.**	OR046547.1	612	OR670010.1	900	*H. perforatum*	Stem
***Chaetomium subglobosum***	OR046549.1	571	OR670012.1	768	*H. perforatum*	Root
***Aspergillus flavipes***	OR046550.1	586	OR670013.1	791	*H. perforatum*	Root
***Cladosporium antarcticum***	OR046553.1	546	OR670016.1	780	*H. helianthemoides*	Root
***Aureobasidium* sp.**	OR046554.1	588	OR670017.1	728	*H. helianthemoides*	Root
***Purpureocillium lilacinum***	OR046557.1	569	OR670020.1	735	*H. helianthemoides*	Root
***Dactylonectria alcacerensis***	OR046558.1	534	OR670021.1	703	*H. scabrum*	Root
**Phoma macrostoma**	OR046562.1	541	OR670025.1	717	*H. scabrum*	Stem

^a^ Abbreviations: ITS, internal transcribed spacer; LSU, large subunit rRNA; NCBI, National Center for Biotechnology Information.

Notably, *A. angustiovoidea* was the only Alternaria species detected in both *H. helianthemoides* and *H. scabrum*. *F. avenaceum* was consistently isolated from the roots of all three host species (Table 1). *A. angustiovoidea* was also recovered from *Hypericum* spp., occurring in the roots of *H. perforatum* and *H. scabrum* and in the stems of *H. helianthemoides*. The colonization pattern of *F. tricinctum* was slightly variable, as it was present in both roots and stems of *H. scabrum* but was detected only in *H. perforatum* among the other species (Table 1; Figure 5). Phylogenetic analysis based on ITS and LSU sequences revealed a high degree of genetic similarity among isolates of the same species from different hosts. Certain fungi, including *A. cerealis*, *A. sorghi*, Absidia sp., *Chaetomium subglobosum*, and *A. flavipes*, were uniquely identified in *H. perforatum*. The number of endophyte species with host-specific colonization was reduced by 20% in *H. helianthemoides* and by 60% in *H. scabrum* relative to *H. perforatum*. In addition, *F. oxysporum*, *Cladosporium antarcticum*, Aureobasidium sp., and Purpureocillium lilacinum were exclusive to *H. helianthemoides*, whereas *Dactylonectria alcacerensis* and Phoma macrostoma were restricted to *H. scabrum*.

### 4.2. Host-Dependent Antibacterial Activity and Minimum Inhibitory Concentration of Endophytic Fungi

The antimicrobial activities of the fungal isolates varied notably, even within the same species obtained from different *Hypericum* hosts, as summarized in Table 2. For example, *A. angustiovoidea* derived from *H. helianthemoides* and *H. scabrum* demonstrated inhibitory effects against all tested bacterial strains, whereas the extract from *A. angustiovoidea* isolated from *H. perforatum* was effective only against *Bacillus subtilis*. Interestingly, the *A. angustiovoidea* extract from *H. perforatum* exhibited the lowest MIC values against *S. aureus*, *S. epidermidis*, and *B. subtilis* compared with the other host-derived isolates (Table 2).

**Table 2.** Minimum Inhibitory Concentration (MIC) of Susceptibility of Different Microbial Strains to Applied Endophytic Fungal Extracts Under In Vitro Conditions ^a^

Antimicrobial Agent	Isolate Code	*Staphylococcus aureus*	*S. epidermidis*	*Bacillus subtilis*	*Pseudomonas aeruginosa*	*Escherichia coli*	Control (+)	Control (-)
***Dactylonectria alcacerensis***	HS-1	n.a.	n.a.	n.a.	n.a.	n.a.	n.a.	wmg
**Alternaria angustiovoidea**	HS-3	6.25	6.25	3.125	100	100	n.a.	wmg
***Fusarium avenaceum***	HS-4	31.2	31.2	62.5	n.a.	n.a.	n.a.	wmg
***F. tricinctum***	HS-5	62.5	31.2	31.2	n.a.	n.a.	n.a.	wmg
**Phoma macrostoma**	HS-6	100	n.a.	100	n.a.	n.a.	n.a.	wmg
***F. tricinctum***	HS-8	62.5	31.2	31.2	n.a.	n.a.	n.a.	wmg
**Alternaria cerealis**	HP-1	25	25	6.25	n.a.	n.a.	n.a.	wmg
***F. avenaceum***	HP-2	n.a.	n.a.	n.a.	n.a.	n.a.	n.a.	wmg
***A. sorghi***	HP-3	n.a.	n.a.	n.a.	n.a.	n.a.	n.a.	wmg
**Absidia sp.**	HP-4	n.a.	n.a.	n.a.	n.a.	n.a.	n.a.	wmg
***F. tricinctum***	HP-5	n.a.	n.a.	n.a.	n.a.	n.a.	n.a.	wmg
***Chaetomium subglobosum***	HP-6	3.125	3.125	3.125	n.a.	100	n.a.	wmg
***Aspergillus flavipes***	HP-7	100	n.a.	100	n.a.	n.a.	n.a.	wmg
***A. angustiovoidea***	HP-8	n.a.	n.a.	50	n.a.	n.a.	n.a.	wmg
***F. oxysporum***	HH-1	n.a.	n.a.	50	n.a.	n.a.	n.a.	wmg
***Cladosporium antarcticum***	HH-3	n.a.	n.a.	n.a.	n.a.	n.a.	n.a.	wmg
**Aureobasidium sp.**	HH-4	n.a.	n.a.	n.a.	n.a.	n.a.	n.a.	wmg
***A. angustiovoidea***	HH-5	100	100	25	100	100	n.a.	wmg
***F. avenaceum***	HH-6	n.a.	n.a.	n.a.	n.a.	n.a.	n.a.	wmg
**Purpureocillium lilacinum**	HH-8	50	50	50	100	100	n.a.	wmg
**Cloxacillin**		0.39	0.39	0.39			n.a.	wmg
**Tobramycin**					6.25	6.25	n.a.	wmg

^a^ Abbreviations: n.a., not active (MIC > 100 μg/mL); wmg, without microbial growth.

Similarly, *F. tricinctum* obtained from *H. scabrum* showed antimicrobial activity against *S. aureus*, *S. epidermidis*, and *B. subtilis*, whereas the corresponding isolate from *H. perforatum* did not exhibit any antibacterial effects. Among *F. avenaceum* isolates, only the extract from *H. scabrum* demonstrated inhibitory activity against these three Gram-positive bacteria, with no activity detected for isolates sourced from *H. helianthemoides* or *H. perforatum*. Overall, 54.55%, 45.46%, and 63.64% of the fungal extracts inhibited *S. aureus*, *S. epidermidis*, and *B. subtilis*, respectively. Notably, *C. subglobosum* displayed the strongest antibacterial effect, with an MIC of 3.125 μg/mL against all tested bacteria. The number of fungal extracts exhibiting activity against Gram-negative bacteria was lower. Only 13.63% of the extracts, including *A. angustiovoidea* and *P. lilacinum*, inhibited *P. aeruginosa*, all with an MIC of 100 μg/mL. Similarly, antibacterial activity against *E. coli* was observed in only 18.18% of the extracts, comprising *A. angustiovoidea*, *P. lilacinum*, and *C. subglobosum*, also with MICs of 100 μg/mL.

### 4.3. Variation in Minimum Bactericidal Concentration of Endophytic Fungi Across Different Host Plants

The MBC of fungal extracts displayed considerable variation, even among isolates of the same endophytic species obtained from different *Hypericum* hosts. The extract of *A. angustiovoidea* derived from *H. perforatum* showed no bactericidal activity, whereas extracts from *A. angustiovoidea* isolated from *H. helianthemoides* and *H. scabrum* demonstrated MBC-associated antimicrobial effects against *S. aureus*, *S. epidermidis*, *B. subtilis*, and *P. aeruginosa*. Among these, the extract from *H. scabrum* exhibited the lowest MBC values against *S. aureus*, *S. epidermidis*, and *B. subtilis* compared with the other isolates.

All *Fusarium* endophyte extracts obtained from *Hypericum* species lacked bactericidal activity against *S. aureus*, *S. epidermidis*, *B. subtilis*, *P. aeruginosa*, and *E. coli*. Overall, 36.36% and 31.81% of the fungal extracts showed MBC-associated activity against *S. aureus* and *S. epidermidis*, respectively, with *C. subglobosum* exhibiting the lowest MBC at 3.125 μg/mL. Approximately 40.9% of the extracts demonstrated bactericidal effects against *B. subtilis*, with both *A. angustiovoidea* and *C. subglobosum* showing the lowest and identical MBC of 3.125 μg/mL. Only *A. angustiovoidea* and *P. lilacinum* extracts displayed MBC-associated activity against *P. aeruginosa*, whereas none of the tested extracts showed bactericidal effects against *E. coli* (Table 3).

**Table 3.** Minimum Bactericidal Concentration (MBC) of Susceptibility of Different Microbial Strains to Applied Endophytic Fungal Extracts Under In Vitro Conditions ^a^

Antimicrobial Agent	Isolate Code	*Staphylococcus aureus*	*Staphylococcus epidermidis*	*Bacillus subtilis*	*Pseudomonas aeruginosa*	*Escherichia coli*	Control (+)	Control (-)
***Dactylonectria alcacerensis***	HS-1	n.a.	n.a.	n.a.	n.a.	n.a.	n.a.	wmg
**Alternaria angustiovoidea**	HS-3	6.25	6.25	3.125	100	n.a.	n.a.	wmg
***Fusarium avenaceum***	HS-4	n.a.	n.a.	n.a.	n.a.	n.a.	n.a.	wmg
***F. tricinctum***	HS-5	n.a.	n.a.	n.a.	n.a.	n.a.	n.a.	wmg
**Phoma macrostoma**	HS-6	n.a.	n.a.	100	n.a.	n.a.	n.a.	wmg
***F. tricinctum***	HS-8	n.a.	n.a.	n.a.	n.a.	n.a.	n.a.	wmg
**Alternaria cerealis**	HP-1	25	25	6.25	n.a.	n.a.	n.a.	wmg
***F. avenaceum***	HP-2	n.a.	n.a.	n.a.	n.a.	n.a.	n.a.	wmg
***A. sorghi***	HP-3	n.a.	n.a.	n.a.	n.a.	n.a.	n.a.	wmg
**Absidia sp.**	HP-4	n.a.	n.a.	n.a.	n.a.	n.a.	n.a.	wmg
***F. tricinctum***	HP-5	n.a.	n.a.	n.a.	n.a.	n.a.	n.a.	wmg
***Chaetomium subglobosum***	HP-6	3.125	3.125	3.125	n.a.	n.a.	n.a.	wmg
***Aspergillus flavipes***	HP-7	100	n.a.	100	n.a.	n.a.	n.a.	wmg
***A. angustiovoidea***	HP-8	n.a.	n.a.	n.a.	n.a.	n.a.	n.a.	wmg
***F. oxysporum***	HH-1	n.a.	n.a.	n.a.	n.a.	n.a.	n.a.	wmg
***Cladosporium antarcticum***	HH-3	n.a.	n.a.	n.a.	n.a.	n.a.	n.a.	wmg
**Aureobasidium sp.**	HH-4	n.a.	n.a.	n.a.	n.a.	n.a.	n.a.	wmg
***A. angustiovoidea***	HH-5	100	100	25	100	n.a.	n.a.	wmg
***F. avenaceum***	HH-6	n.a.	n.a.	n.a.	n.a.	n.a.	n.a.	wmg
**Purpureocillium lilacinum**	HH-8	50	50	50	100	n.a.	n.a.	wmg
**Cloxacillin**		0.39	0.39	0.39			n.a.	wmg
**Tobramycin**					6.25	6.25	n.a.	wmg

^a^ Abbreviations: n.a., not active (MIC > 100 μg/mL); wmg, without microbial growth.

## 5. Discussion

Endophytic fungi were isolated from these plants and identified, with most belonging to the phylum Ascomycota, except for Absidia sp., which was assigned to Zygomycota. Previous studies indicate that Ascomycota dominates endophytic communities (84%), followed by Basidiomycota (10%), Mucoromycota (5%), and Oomycota (1%) (18). Members of Ascomycota and a substantial proportion of Basidiomycota are commonly associated with plants as saprobes, parasites, or symbionts, and Ascomycota endophytes frequently colonize root tissues both intracellularly and intercellularly (19). The broad phylogenetic distribution of plant-associated fungi, supported by fossil evidence, suggests a long-standing evolutionary reliance of Ascomycota on living or dead plants for nutritional support since the early stages of their evolution (20). The genus Absidia is traditionally recognized as an opportunistic pathogen responsible for rhinocerebral mycoses and primary cutaneous, pulmonary, or gastrointestinal infections, particularly in immunocompromised individuals, burn victims, or patients with diabetes or traumatic injuries (21). In addition to its clinical significance, Absidia is of interest for potential biotechnological applications (22). However, there are no prior reports of this genus exhibiting endophytic behavior. Accordingly, this study provides the first evidence of Absidia sp. functioning as an endophyte within the stem tissue of *H. perforatum*.

The primary endophytic fungal species identified in the current study belonged to the genera Alternaria and *Fusarium*. Consistent with our findings, previous investigations have demonstrated the presence of Alternaria fungi across various *Hypericum* species. For instance, Samaga and Rai (9) documented the endophytic behavior of *A. brassicae* in *H. mysorense*, although this was the only study to identify Alternaria at the species level. Other studies have corroborated the presence of *Alternaria* spp. as endophytes in *H. perforatum*, *H. humifusum*, *H. monogynum*, *H. erectum*, and *H. japonicum*, although only at the genus level (7, 8, 23). Notably, the present research introduces new reports of *A. cerealis*, *A. sorghi*, and *A. angustiovoidea* as endophytes within *H. perforatum*, *H. helianthemoides*, and *H. scabrum*, for which previous documentation is lacking. Regarding *Fusarium* fungi, our results align with existing literature indicating the presence of *F. oxysporum* in *H. humifusum*, *H. erectum*, and *H. monogynum* (7). However, there is no prior report documenting the endophytic behavior of *F. avenaceum* and *F. tricinctum* in *Hypericum* plants. Furthermore, although *C. subglobosum* has not been directly reported in *Hypericum* plants, Samaga and Rai (9) described the endophytic behavior of the closely related species *C. globosum* in *H. mysorense*. Similarly, Henzelyová et al. (7) identified *A. pullulans* in *H. erectum* and Dactylonectria sp. in *H. canariense*, whereas our study isolated Aureobasidium sp. and *D. alcacerensis* as endophytes from *H. helianthemoides* and *H. scabrum*, respectively. Whether these unidentified species correspond to those reported by Henzelyová et al. (7) requires further investigation. Notably, our research identified *A. flavipes* as an endophyte in *H. perforatum*, whereas the literature reports different species, such as *A. calidoustus*, *A. ustus*, *A. nidulans*, and A. versicolor, from *H. mysorense* (9). In addition, *A. flavus* and an unknown *Aspergillus* sp. have been reported from *H. japonicum* and *H. perforatum*, respectively (23). Phoma sp. was identified as an endophyte of *H. mysorense* only in the study by Samaga and Rai (9), whereas our investigation reported *P. macrostoma* from *H. scabrum*. Furthermore, our study identified *C. antarcticum* from *H. helianthemoides*, a species not previously reported as an endophyte in this specific *Hypericum* species, although related species such as *C. herbarum*, *C. oxysporum*, and *C. sphaerospermum* have been documented in *H. mysorense* and *H. japonicum* by Samaga and Rai (9). Moreover, an unidentified *Cladosporium* sp. has been reported from *H. kouytchense* in the study by Henzelyová et al. (7). To the best of our knowledge, all endophytes identified in the current study represent new records for the investigated plant species.

Our findings indicate that only 60% of the identified endophytic isolates obtained from *Hypericum* plants exhibited antimicrobial activity. Notably, all *Fusarium* spp. demonstrated antimicrobial activity. Consistent with our observations, existing research has characterized 50 secondary metabolites derived from *Fusarium*, showing diverse bactericidal effects, particularly against Gram-positive bacteria (24). These metabolites include fusariumins C and D, enniatins, beauvericin A, trichosetin, enantiomers, lateritin, naphthoquinones, linoleic acid, epi-equisetin, fusaroxazin, neomangicol B, fungerin, and fusariumnols A and B (25). In addition, butenolide, nine 2-pyrrolidone, lucilactaene analogs, karimunones B, and Fusapyridon A, identified as *Fusarium* mycotoxins, exhibited selective inhibitory activity against Gram-negative bacteria (24). Furthermore, our investigation revealed antibacterial activity in endophytic Alternaria species. A review of the literature supports our findings, indicating that Alternaria spp. produce various antibacterial metabolites, such as tenuazonic acid, altersetin, pyrophen, brassicicolin A, herbarin A, macrosporin, hydroxybostrycin, and altersolanols, all of which exhibit strong antimicrobial activity (26). Other endophytic isolates demonstrating antimicrobial properties included *P. macrostoma*, *C. subglobosum*, *A. flavipes*, and *P. lilacinum*. Recent chemical studies on *A. flavipes* have led to the discovery of numerous molecules with novel structures and notable biological activities. These include structurally complex merocytochalasans, lumazine peptides with significant antibacterial and NF-κB inhibitory activities, chlorinated PKS-NRPS hybrid metabolites with potent pancreatic lipase inhibitory activity, and rare prenylated phenylbutyrolactones (27). The metabolite spectrum encompasses bioactive compounds such as phomenon, phomin, phomodione, cytochalasins, cercosporamide, phomazines, and phomapyrone reported from *P. macrostoma* and other Phoma spp., exhibiting a broad range of activities, including antimicrobial, antiviral, antinematode, and anticancer properties (28). Moreover, kojic acid, ethyl acetate, and hydroxybenzoic acids derived from *P. lilacinum* have been reported to possess antibacterial activities (29). Although the antibacterial activity of the *C. subglobosum* extract was observed in our study, it has not been explicitly documented in the available literature. Consequently, our research, consistent with prior studies, underscores the strong association between the antibacterial properties of the identified endophytes and their secondary metabolites, emphasizing the need for meticulous identification in future investigations.

### 5.1. Conclusions

The present investigation examined *Hypericum* species in the Quchan region of Iran, focusing on their associated endophytic fungi. A range of fungal taxa were identified, including the notable discovery of Absidia sp. in *H. perforatum*, providing new insights into plant-fungal interactions. The study also documented the presence of Alternaria spp., *Fusarium* spp., and additional endophytes exhibiting antimicrobial activity. The observed antimicrobial effects were closely linked to the production of bioactive secondary metabolites, highlighting their potential applications in biotechnology. These results expand current understanding of the endophytic ecology of *Hypericum* species and underscore the importance of further studies to elucidate the ecological functions and biotechnological potential of the newly identified endophytic fungi.

## Data Availability

The raw datasets generated during the current study are available from the corresponding author upon reasonable request.
